# Development of Ultrasound-Assisted Extraction to Produce Skin-Whitening and Anti-Wrinkle Substances from Safflower Seed

**DOI:** 10.3390/molecules27041296

**Published:** 2022-02-15

**Authors:** Suh-Hee Yeom, Da-Hye Gam, Jun-Hee Kim, Jin-Woo Kim

**Affiliations:** 1 Department of Food Science, Sun Moon University, Natural Science 118, 70 Sunmoon-ro 221, Tangjeong-myeon, Asan-si 336-708, Korea; yshee0017@naver.com (S.-H.Y.); ank7895@naver.com (D.-H.G.); jun981014@naver.com (J.-H.K.); 2 FlexPro Biotechnology, Natural Science 128, 70 Sunmoon-ro 221, Tangjeong-myeon, Asan-si 336-708, Korea

**Keywords:** safflower seed, skin-whitening, anti-wrinkle, maleic acid, levulinic acid, ultrasound-assisted extraction, optimization

## Abstract

In this study, ultrasound-assisted extraction (UAE) was applied to extract bioactive substances with skin-whitening, anti-wrinkle, and antioxidant effects from safflower seeds, and the extraction conditions were optimized by a central composite design. The independent variables, including extraction time (5.0~55.0 min), extraction temperature (26.0~94.0 °C), and ethanol concentration (0.0~100%), were optimized to increase tyrosinase activity inhibitory (TAI), collagenase activity inhibitory (CAI), and radical scavenging activity (RSA), which are indicators of skin-whitening, anti-wrinkle, and antioxidant effects. An extraction time of 26.4 min, extraction temperature of 52.1 °C, and ethanol concentration of 50.7% were found to be optimum conditions of UAE, under which TAI, CAI, and RSA were 53.3%, 91.5%, and 27.7%, respectively. The extract produced by UAE was analyzed by LC-MS/MS, and maleic acid and levulinic acid were identified as the main substances. Therefore, UAE is evaluated as an effective process to extract skin-whitening, anti-wrinkle, and antioxidant substances from safflower seeds at lower temperatures and shorter extraction times compared to the conventional extraction methods. Overall, safflower seeds extract can be used as a material for value-added cosmetics, including maleic acid and levulinic acid, which have bioactive functions.

## 1. Introduction

Recently, as living standards have improved and interest in health and beauty has increased, the demand for functional cosmetics that not only improve skin health and beauty but also delay skin aging is increasing rapidly [1,2]. Consumer preferences for functional cosmetics have led to changes such that convenience and functionality are emphasized and new types of cosmetics, such as multifunctional cosmetics, are increasingly being preferred [3]. Therefore, the research and development of cosmetic substances that have multiple functions, such as skin-whitening, anti-wrinkle, and antioxidant, are increasing [4]. Skin-whitening products are mainly composed of substances that inhibit melanin synthesis, which is the cause of pigmentation-related pathways such as freckles, age spots, dark spots, etc. Particularly, cosmetics that contain inhibitors of tyrosinase, a major enzyme involved in melanin synthesis, are being actively developed, and various tyrosinase inhibitors have been tested in cosmetics and pharmaceuticals for preventing the overproduction of melanin in epidermal layers [5,6]. Collagenase is a type of matrix metalloproteinase (MMP) that can hydrolyze peptide bonds in macromolecules such as aggrecan, elastin, fibronectin, selectin, gelatin, laminin, and collagen, which decompose the dermal matrix and increases wrinkles by reducing skin elasticity [7]. The research for the development of anti-wrinkle agents that primarily focuses on the inhibitory mechanism of collagenase is also being conducted to inhibit the degradation of collagen, a protein responsible for skin tension, strength, and elasticity [8]. Therefore, the substances that inhibit collagenase activity may have beneficial effects to maintain healthy skin by preventing dermal matrix degradation [9].

Melanin and wrinkle production are known to be directly or indirectly related to reactive oxygen species (ROS) [10]. ROS promote the process of tyrosine oxidation into dopaquinone, thus accelerating melanin synthesis, therefore, it is important to use a substance that removes ROS for the downregulation of tyrosinase activity [11,12]. It is known that ROS induces oxidative stress in skin cells and increases collagenase activity by oxidation of the phospholipid membrane and disruption of the transmembrane signaling pathway, thereby degrading the structure of collagen and resulting in the formation of wrinkles and sagging skin and reducing skin elasticity [13]. Therefore, for effective skin-whitening and anti-wrinkling, it is important to discover antioxidant substances that simultaneously inhibit tyrosinase and collagenase activities, as well as effectively remove ROS, which leads to melanin synthesis and collagen degradation [14].

Although kojic acid, albumin, and vitamins are widely used as skin-whitening agents that inhibit melanin synthesis, they have a low whitening effect on the excessively produced melanin and can also cause contact dermatitis, sensitization, and erythema [15,16]. In addition, there are many limitations on using them as a cosmetic substance due to their long-term storage stability [17]. Synthetic antioxidants such as butylated hydroxyanisole (BHA), butylated hydroxytoluene (BHT), and tert-butylhydroquinone (TBHQ) have high antioxidant effects and are being widely used in the food, cosmetic, and pharmaceutical industries; however, they have limitations related to safety, and adverse effects, such as fat denaturation, liver toxicity, and carcinogenicity, caused by their excessive use have been reported [18,19]. Moreover, these antioxidants have disadvantages in stability compared to natural antioxidants, because thermal stability is reduced during the heating process, and their antioxidant effect is reduced. Therefore, to replace those currently in use, there is an urgent need to identify antioxidants from natural sources that have both skin-whitening and anti-wrinkle properties and cause minimal adverse effects.

Safflower, *Carthamus tinctorius*, is a highly branched biennial plant that belongs to the Asteraceae and is widely grown in Asia, East Africa, and North America [20]. Traditionally, its flowers and seeds are widely used for food or medicinal purposes, and safflower are now commercially cultivated to produce moisturizers and cooking oil [20,21]. Safflower seed oil is absorbed quickly into the skin, making it an excellent moisturizing facial oil suitable for all skin types [22]. In particular, it contains large amounts of levulinic acid, an essential fatty acid that works as both an analgesic and antioxidant [23]. Recently, safflower seed oil has been widely used in the food and cosmetics industries, safflower seed extract (SSE) is also used and comparatively in various ways. Several studies have suggested that SSE protects biological tissue owing to their fracture-healing efficacy and is used for treating osteoporosis, as well as protecting the liver [24,25]. Several studies have evaluated the functionality of SSE in the cosmetic industry; however, studies exploring the main substances in SSE are limited. Thus, in order to increase the utilization of safflower seeds as functional cosmetics, in-depth research focusing on substances present in SSE is required. The goal of this study was to maximize the extraction of useful substances from safflower seeds by applying UAE and to identify the main substances in SSE, along with the evaluation of the whitening and anti-wrinkle properties, to increase the industrial applicability of safflower seeds.

## 2. Results

### 2.1. Model Fitting

The effects of various UAE conditions on SSE’s tyrosinase activity inhibition (TAI) and collagenase activity inhibition (CAI) and radical scavenging activity (RSA) were evaluated to discover multifunctional components with skin-whitening, anti-wrinkle, and antioxidant properties. Based on the central composite design (CCD), one of the statistical optimization methodologies, UAE conditions, including extraction time (5.0~55.0 min), extraction temperature (26.0~94.0 °C), and ethanol concentration (0.0~100%), were set as the independent variables. The range of individual independent variables used in the CCDs was established based on previous studies conducted in our laboratory [26]. The experimental values obtained for 17 experimental conditions are presented in Table 1. When changes in the independent variables according to different extraction conditions were identified, the maximum TAI value was found to be 63.5% at an extraction time of 15.0 min, extraction temperature of 40.0 °C, and ethanol concentration of 80.0%, while the maximum value of the CAI was 96.9% at an extraction time of 45.0 min, extraction temperature of 40.0 °C, and ethanol concentration of 80.0%. At an extraction time of 30.0 min, extraction temperature of 60.0 °C, and ethanol concentration of 50.0%, the RSA was the highest at 31.9%.

A quadratic regression equation was deduced to predict the TAI, CAI, and RSA based on 17 experimental values from CCD, and R^2^ was used to evaluate the suitability of the predicted experimental values. When R^2^ is closer to 1, the values predicted by the quadratic regression equation are evaluated to be closer to the experimental values. The R^2^ of the quadratic regression equations for the TAI, CAI, and RSA deduced based on the experimental values were 0.8823, 0.9330, and 0.9233, respectively, indicating that the experimental and predicted values were highly consistent. In addition, as a result of the analysis of variance, *p*-values showing the statistical significance of TAI, CAI, and RSA were found to be 0.0149, 0.0024, and 0.0038, respectively; the significance level was lower than 0.05, indicating the significance of correlation between the dependent and independent variables (Table 2). The significance evaluation also indicated that the ethanol concentration had the most significant effect on the TAI, CAI, and RSA among the independent variables (Table 3). These data indicate that ethanol is a major independent variable in the optimization of the UAE process and should be considered a priority for optimization.

The experimental values of the TAI, CAI, and RSA, according to the 17 extraction conditions, were set by independent variables according to coded levels: X_1_: extraction time, X_2_: extraction temperature, X_3_: ethanol concentration, tyrosinase activity inhibitory (TAI), collagenase activity inhibitory (CAI), and radical scavenging activity (RSA).

### 2.2. Optimization of UAE Condition for Maximizing TAI

**Figure 1:**
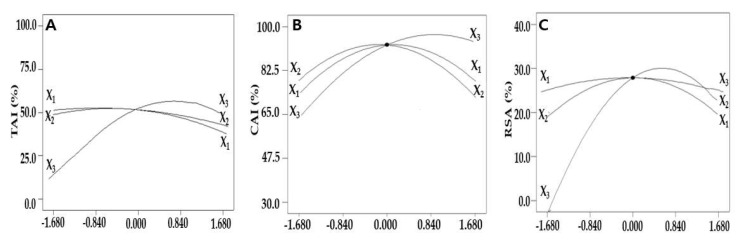
Perturbation plots for the evaluation of the effect of three independent variables, including the extraction time (X_1_), extraction temperature (X_2_), and ethanol concentration (X_3_) on the TAI (**A**), CAI (**B**), and RSA (**C**) of safflower seeds.

To predict the UAE conditions that maximize the TAI extraction, it was identified how the values of a single independent variable affected the TAI using a perturbation plot in which the maximum and minimum points of TAI can be visualized. When the variation rates of TAI were compared according to the value of an independent variable, the TAI increased greatly when the ethanol concentration increased, whereas changes in the TAI according to extraction time and extraction temperature were insignificant compared to the ethanol concentration, which was evaluated to affect the dependent variables the most; this was consistent with the previous ANOVA results (Figure 1A).

**Figure 2:**
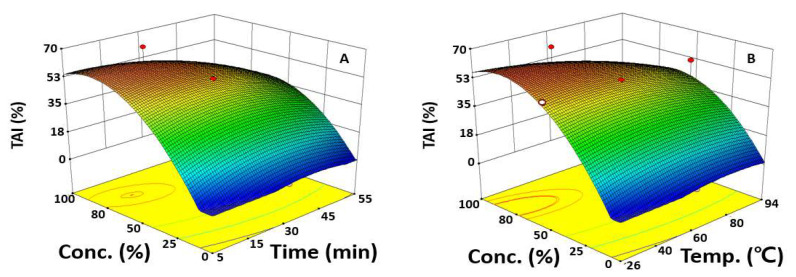
Response surface plots represent the effect of two variables on the TAI. Each response surface plot depicts the influence of two variables on the TAI, while the third variable was fixed at the center point of 30.0 min or 60.0 °C. TAI as a function of extraction time and ethanol concentration (**A**), extraction temperature and ethanol concentration (**B**).

To evaluate the interaction of independent variables on the response, the other variable was fixed based at the center point, a coded value of zero, and the remaining independent variables were changed. When the interactive effect of the extraction time and ethanol concentration on the TAI was evaluated by fixing the extraction temperature to 60.0 °C, the TAI decreased slightly after the maximum at extraction time 33.5 min (Figure 2A). There was a rapid increase in the TAI as the ethanol concentration increased, reconfirming that the ethanol concentration significantly affected the TAI compared to the extraction time. When the effects of the extraction temperature and ethanol concentration on the TAI were evaluated at a fixed extraction time of 30.0 min, the maximum value was shown at an extraction temperature of 29.1 °C and ethanol concentration of 79.1%, and the change in the TAI was highly dependent on the ethanol concentration, confirming that the ethanol concentration significantly affected the TAI compared to the extraction temperature (Figure 2B). These data correspond to the results of a study that compared the bioactivities of *Artemisia annua* L. extracts, which showed that the higher the ethanol concentration, the higher the TAI value can be obtained [27]. In addition, in the study of Han et al., the TAI was found to be 19.6% in the extraction of safflower seeds using 70% ethanol at 60 °C, showing a lower value than the TAI of 63.5% in this study, indicating that the optimization of the extraction condition is essential in maximizing the TAI [28].

The extraction with 100% ethanol led to a >50.0% inhibition of TAI, which was four times more TAI compared to when the extraction was performed using water. A hypothesis is that the change in solvent polarity with higher ethanol concentrations increases the extraction of the antioxidant effect from safflower seeds. Another is that, as the surface tension and viscosity of a solvent decreases when the ethanol concentration increases, the solvent more easily infiltrates the cell wall, which increases the extraction efficiency. Therefore, to extract bioactive substances from safflower seeds, optimizing the ethanol concentration should be prioritized to increase the extraction efficiency of skin-whitening substances.

### 2.3. Optimization of UAE Condition for Maximizing CAI

To optimize the extraction conditions by evaluating the effect of the independent variables on the CAI, two variables were fixed at the center point, and a one-factor-at-a-time experiment that visualized the effect of a single variable on the CAI was conducted (Figure 1B). As the extraction time, extraction temperature, and ethanol concentration increased, the CAI also increased and then began to decrease after reaching a maximum at an extraction time of 30.0 min and extraction temperature of approximately 60.0 °C. As the ethanol concentration increased, the CAI also increased and reached a maximum at an ethanol concentration of approximately 80.0%, which was similar to the preceding TAI results.

**Figure 3:**
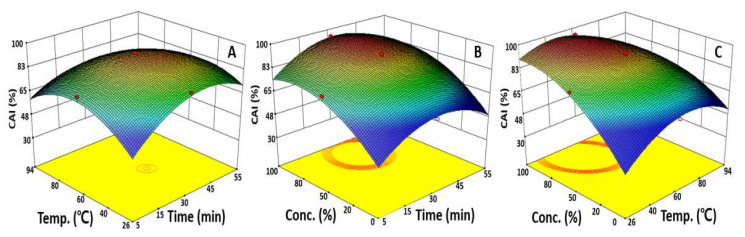
Response surface plots represent the effect of two variables on the CAI. Each response surface plot depicts the influence of two variables on the CAI, while the third variable was fixed at the center points of 30.0 min, 60.0 °C, and 50.0%. CAI as a function of extraction time and extraction temperature (**A**), extraction time and ethanol concentration (**B**), extraction temperature and ethanol concentration (**C**).

To identify a reciprocal action between variables, the effect of the changes in two independent variables on the CAI was presented using a response surface plot. The CAI had a maximum value of 93.0% at an extraction time of 32.8 min, extraction temperature of 56.2 °C, and was fixed at a 50.0% ethanol concentration (Figure 3). Then, the CAI value decreased after the peak extraction time and extraction temperature were reached to derive the condition for optimal extraction. In terms of changes in the CAI according to the extraction time and ethanol concentration, the highest CAI value was obtained at 14.9 min of extraction time and 77.2% ethanol; after which, it decreased. A similar trend can be found in Figure 3C presenting the correlation between the ethanol concentration and extraction temperature. With a fixed extraction time of 30.0 min, the CAI increased as the extraction temperature and ethanol concentration increased to a maximum at 51.3 °C and 81.9% ethanol, respectively, and then rapidly decreased. The response surface plot predicted the optimal UAE condition as an extraction time of 45.0 min, extraction temperature of 40.0 °C, and ethanol concentration of 50.0%. In addition, the CAI obtained through the statistically based optimization in this study was about four times higher than that of the CAI identified in hot water extraction, indicating that the UAE optimization was effective in increasing the extraction efficiency [29]. This is similar to the results of a study on *Polygala japonica* Houtt extract, showing that the ethanol concentration affects the CAI the most, and the maximum value of the CAI was 78.0% in an extraction using 50.0% ethanol [30]. These data suggest that optimizing the ethanol concentration as a priority will be the most effective way to increase the extraction of bioactive substances with anti-wrinkle properties from safflower seeds.

### 2.4. Optimization of UAE Condition for Maximizing RSA

To evaluate the effect of a single independent variable on the RSA, an indicator of the antioxidant effect, in the extraction of bioactive substances from safflower seeds, two variables were fixed, and the transition of the dependent variables was plotted. As the extraction time, extraction temperature, and ethanol concentration increased, the RSA also increased to a certain level, but after reaching the maximum value at the extraction time of 30.1 min, extraction temperature of 60.2 °C, and ethanol concentration of 76.5%, it tended to decrease again, as in the previous TAI. The ethanol concentration affected the RSA the most, whereas the extraction time and extraction temperature had insignificant effects (Figure 1C).

**Figure 4:**
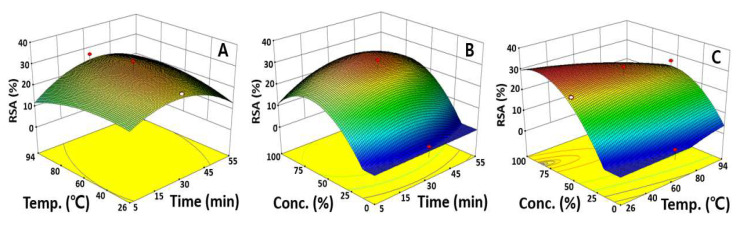
Response surfaces plots represent the effect of two variables on the RSA. Each response surface plot depicts the influence of two variables on the RSA, while the third variable was fixed at the center points of 30.0 min, 60.0 °C, and 50.0%. RSA as a function of extraction time and extraction temperature (**A**), extraction time and ethanol concentration (**B**), extraction temperature and ethanol concentration (**C**).

To evaluate the intervariable influences, one independent variable was fixed at its center point, and the changes in the RSA were visualized using a response surface plot according to the fluctuation rates. In Figure 4A, showing a response surface plot that was fixed at 50.0% ethanol, changes in the RSA were not significant as the extraction time and temperature increased, which was consistent with the previous results regarding the significance of the independent variables. In terms of the effect of extraction time and ethanol concentration on the RSA, the maximum RSA value of 30.3% was predicted at 31.5 min of extraction time and 68.0% ethanol concentration, which depicts the low tendency for changes in the RSA based on the extraction time compared the ethanol concentration.

A maximum RSA of 32.1% was also predicted at a 26.3 °C extraction temperature and 81.5% ethanol concentration through a response surface plot presenting the effects of the extraction time and ethanol concentration, confirming again that the ethanol concentration significantly affects the RSA, as indicated by the ANOVA results (Figure 4). These data are similar to studies showing that up to 81.5% of the RSA was obtained when an antioxidant was extracted using 95.0% ethanol from *Dioscorea polystachya* and also consistent with Han’s study, which reported a maximum RSA of 76.0% when 80.0% ethanol was used to extract antioxidants from *Cicer arietinum* [31,32]. Therefore, it was found that bioactive materials, especially antioxidants, are greatly affected by the ethanol concentration in extracting useful substances from various plants. The antioxidant activity was measured as a control group using ascorbic acid, and the value was measured as 90.3%. The sentence was newly inserted. The ANOVA and response surface plot results indicated that the dependency on the ethanol concentration was high compared to the extraction time and extraction temperature. A previous study reported that UAE increases the rate of mass transfer due to cavitation forces, which causes the breaking of small vacuum bubbles in the liquid–solid interphase, resulting in localized pressure, accelerating the rupture of plant tissues and the release of intracellular substances into the solvent [33]. Considering all the results together, the UAE proved to be a highly effective extraction process that saves time and cost compared to the traditional extraction techniques. 

### 2.5. Model Validation

**Figure 5:**
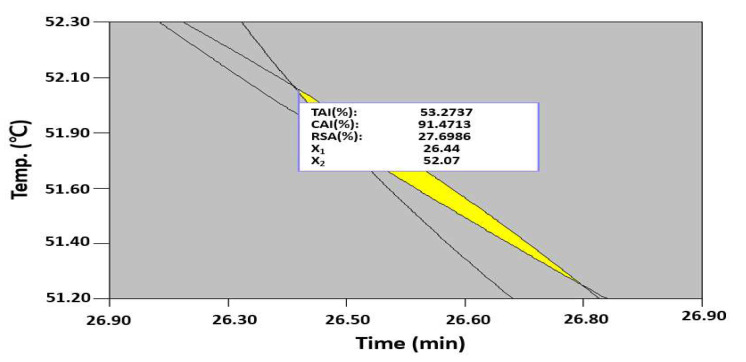
Superimposing contour map for the prediction of optimized conditions of the TAI, CAI, and RSA from three independent variables, including the extraction time, extraction temperature, and ethanol concentration. X_1_; extraction time and X_2_; extraction temperature.

To extract multifunctional, bioactive substances with skin-whitening, anti-wrinkle, and antioxidant properties from safflower seeds, the maximization conditions were identified to predict the optimal UAE conditions by superimposing the contour plot of each response surface. In the UAE process, the extraction time must be reduced to increase the productivity and reduce the process cost. Thus, when a minimum extraction time of 26.4 min was prioritized to conduct the optimization, the maximum values of the TAI, CAI, and RSA were predicted to be 53.3%, 91.5%, and 27.7%, respectively, at an extraction temperature of 52.1 °C and ethanol concentration of 50.4% (Figure 5). The results of a validation experiment based on these predicted conditions were as follows: TAI of 55.1 ± 1.48%, CAI of 89.1 ± 2.14%, and RSA of 29.2 ± 1.02%, showing similar values as predicted by quadratic regression models, which were found to be effective in predicting the maximum value based on the CCD.

### 2.6. LC-MS/MS Analysis

**Figure 6:**
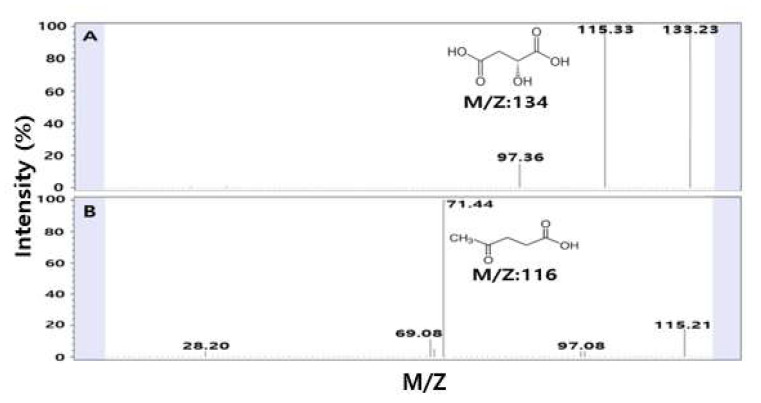
LC-MS/MS chromatogram of the major substances from safflower seeds: (**A**) malic acid (134 *m*/*z*) and (**B**) levulinic acid (116 *m*/*z*).

The optimal conditions for the extraction of bioactive substances from SSE showing high TAI, CAI, and RSA were selected through the above statistical optimizations. When an analysis was performed in the negative mode of LC-MS/MS to detect the substances present in the extracts, two major molecular ion peaks were found at 134 and 116 *m*/*z* (Figure 6). In the negative-ion mode, 134 *m*/*z* corresponds to the molecular formula of malic acid, and 116 *m*/*z* corresponds to the molecular formula of levulinic acid. Based on the molecular weight, it was confirmed that one proton of malic acid was detected in a separate form in the extract, and it was confirmed that H_2_O was removed from malic acid, resulting in the separation of an anion peak of 115 *m*/*z* (Figure 6A). The detection of malic acid in SSE was consistent with the results of a study investigating the bioactive substances of safflower seeds using 60.0% by Kim et al., reporting that malic acid was detected as a major organic acid in SSE [34]. Malic acid is well-known as an antioxidant and is a cosmetic substance primarily used in skin care products helping to improve skin pigmentation and complexion and affecting the formation of skin collagen [35]. Levulinic acid and its derivatives, like sodium levulinate, are used in organic and natural cosmetic substances for skin conditioning, and the pH control agent and bactericidal effects [36,37]. In particular, levulinic acid is known to have excellent antibacterial, acne, and whitening effects, so levulinic acid found in safflower seeds can be used as cosmetic ingredients and antibacterial agents [38]. It stabilizes the formulations and emulsions and is used in cosmetics for its antiseptic properties and also acts as a skin conditioning agent [39]. It is predicted that SSE’s functionality is attributable to levulinic acid and malic acid as the main substances, and SSE is expected to be effective when used as a cosmetic substance.

## 3. Materials and Methods

### 3.1. Materials and Reagents

The safflower seed was purchased from Nonghyup (Cheonan, Korea) in June 2021 and stored at −21 °C. Distilled water and ethanol (Samchun Pure Chemical, Seoul, Korea, 99.0%) were used as the extraction solvents. Aluminum chloride, potassium acetate, 3,4-dihydroxyphenylene (L-DOPA), mushroom tyrosinase, 4-phenylazobenzyloxycarbonyl-Pro-Leu-Gly-Pro-D-Arg (Pz-PLGPR), collagenase from Clostridium histolyticum, and 2,2-diphenyl-picrylhydrazyl (DPPH) were used as the reagent for evaluating skin-whitening and anti-wrinkle effects. Kojic acid and ascorbic acid were used as positive controls in the measurement of the TAI and CAI, and the reagents used in the analysis were purchased from Sigma-Aldrich (St. Louis, MO, USA), and all other reagents used in the extractions and analysis were extra-pure grade.

### 3.2. Ultrasound-Assisted Extraction

In order to the optimization of the UAE conditions of a bioactive substance from safflower seeds, the extraction time (5.0~55.0 min), extraction temperature (26.0~94.0 °C), and ethanol concentration (0.0~100%) were set as independent variables. Ten milliliters of different concentrations of ethanol were added to 1 g of safflower seeds, the UAE was performed using a desktop ultrasound device (SD-250H, Gyeonggi, Korea), centrifuged at 10,000 rpm for 10 min, and the supernatant was taken and frozen for the following experiments. Each independent variable is examined at five levels based on the preliminary single variable analysis and literature data. The levels of the independent variables were coded at five levels according to the equation where x_i_ and x_i_ are the coded and actual values of an independent variable, respectively (Table 4).

(1) $$x_{i}=\frac{X_{i}-X_{0}}{{\Delta X}_{i}}\;i=1,\;2,\;3,\;4$$

X_0_ is the actual value on the center point of X_i_, and ΔX_i_ is the value of the step change.

### 3.3. Measurement of Tyrosinase Activity Inhibitory (TAI)

The TAI was performed as described by Jo et al., and skin-whitening function was confirmed based on the inhibition of tyrosine activity [40]. After mixing 0.4 mL of 67-mM sodium phosphate buffer (pH 6.8) with 0.2 mL of extract and 0.2 mL of 10-mM L-DOPA, mushroom tyrosinase was added, and kojic acid was used as a positive control. The reaction was performed at 25.0 °C for 30 min, and the generated Dopachrome was measured at 475 nm using a spectrophotometer (Optizen 2120 uv, Mecasys Co. Daejeon, Korea), and the TAI was calculated as shown below.

(2) $$\mathrm{TAI}\left(\%\right)=\left\{1-\frac{\mathrm{Absorbance}\;\mathrm{of}\;\mathrm{sample}}{\mathrm{Absorbance}\;\mathrm{of}\;\mathrm{control}}\right\}\times 100$$

### 3.4. Measurement of Collagenase Activity Inhibitory (CAI)

The CAI was performed as described by Gam et al. and evaluated anti-wrinkle effects of the SSE by measuring the collagenase activity inhibition [41]. The solution of the substrate was prepared by mixing 1.2 mg/mL of Pz-PLGPR, 0.4 mg/mL of collagenase, and tris buffer containing 0.1 M of tris and 4 mM of CaCl_2_. The mixture was allowed to react at 37.0 °C for 30 min, and 0.25 mL of citric acid was added for the termination of the reactions. After mixing with 1.2 mL of ethyl acetate, the reaction solution, the supernatant was separated and absorbance measured at 320 nm. The CAI was calculated as a percentage based on the control group.

(3) $$\mathrm{CAI}\left(\%\right)=\left\{1-\frac{\mathrm{Absorbance}\;\mathrm{of}\;\mathrm{sample}}{\mathrm{Absorbance}\;\mathrm{of}\;\mathrm{control}}\right\}\times 100$$

### 3.5. Measurement of Radical Scavenging Activity (RSA)

Measurement of the antioxidant effect of SSE was used by modifying the method of Marxen et al. [42]. The stock solution was prepared by 0.1-M DPPH mixing with methanol. The stock solution was diluted with methanol to have an optical density of 1.0 ± 0.1 at 517 nm using a spectrophotometer. A 1.25 mL DPPH solution was mixed with 0.25 mL SSE and allowed to rest in the dark room for 20 min at room temperature. The antioxidant effect of SSE was calculated as a percentage as shown below.

(4) $$\mathrm{RSA}\left(\%\right)=\left\{1-\frac{\mathrm{Absorbance}\;\mathrm{of}\;\mathrm{sample}}{\mathrm{Absorbance}\;\mathrm{of}\;\mathrm{control}}\right\}\times 100$$

### 3.6. LC-MS/MS Analysis

LC-MS/MS (innigan TSQ Quantum Thermo Fisher Sci., Waltham, MA, USA) was used for the analysis of the major substances in SSE, and the analysis injection volume was 10 μL, and the ROC C18 column (3.0 × 150 mm, RESTEK Co., Bellefonte, PA, USA) was maintained at 30 °C. Mobile phase A (0.1% formic acid in water) and mobile phase B (0.1% formic acid in acetonitrile) were mixed, and the flow rate was set to 0.2 mL/min to perform separation. The gradient conditions of the mobile phase for the LC-MS/MS analysis were the volume ratio of mobile phase A 0.0 to 1.0 min for 95.0%, 11.0 to 14.0 min for 0.0%, 14.0 to 15.0 min for 95.0%, and 15.0 to 20.0 min for 95.0%. The SSE’s chromatographic separation was performed used a negative ion electrospray ionization (ESI), utilizing the turbo ion spray mode. The final setting for the ionization of SSE was as follows: gas temperature, 270 °C; gas flow, 19 L/min; sheath gas temperature, 400 °C; and sheath gas flow, 10 L/min. The main substances in the SSE were analyzed by measuring the mass spectrum of the main substance at 100~800 *m*/*z* and comparing the fragmentation patterns of LC-MS/MS data from the mass library for the standard substances.

## 4. Conclusions

This study optimized the condition of UAE using CCD to produce extracts with maximum skin-whitening, anti-wrinkle, and antioxidant activities from safflower seeds. In order to optimize the UAE conditions, the extraction time, extraction temperature, and ethanol concentration were set as independent variables to predict conditions for simultaneous maximization of the TAI, CAI, and RSA. It was confirmed that the TAI, CAI, and RSA, which are indicators of skin-whitening, anti-wrinkle, and antioxidant activities are respectively greatly affected by the concentration of ethanol used for extracting bioactive substances from safflower seeds using UAE. As the ethanol concentration increased, the amount of bioactive substances extracted initially increased and then decreased after reaching the maximum. Both water and ethanol are hydrophilic solvents and are predicted to be effective solvents for dissolving both acids. Ethanol has a lower viscosity than water, which is expected to facilitate penetration into plant cell wall micropores, and it can be assumed that the extraction efficiency increased due to an increase in the liquid–solid contact and mass transfer. Therefore, this study confirmed that the optimization of ethanol concentration should be prioritized to increase the skin-whitening, anti-wrinkle, and antioxidant activities of safflower seeds. By superimposing each response surface plot, an extraction time of 26.4 min, extraction temperature of 52.1 °C, and ethanol concentration of 50.7% were found to be the optimal UAE conditions; under these conditions, the TAI, CAI, and RSA were maximized to 53.3%, 91.5%, and 27.7%, respectively. In the LC-MS/MS analysis of the main substances of SSE obtained by the optimal UAE conditions, maleic acid and levulinic acid were identified as the main components. An LC-MS/MS analysis of the main substances in SSE was conducted under optimum conditions, which identified malic acid and levulinic acid as main substance. Furthermore, it can be concluded that the main components of such as malic acid and levulinic acid, as well as SSE, are highly likely to be used as multifunctional cosmetics substances with skin whitening, anti-wrinkle, and antioxidant effects.

## Figures and Tables

**Table 1:** Independent variables and responses from 17 experimental on the TAI, CAI, and RSA of SSE by UAE.

Run No.	Extraction Conditions			TAI (%)	CAI (%)	RSA (%)
	X_1_	X_2_	X_3_			
1	15.0	40.0	20.0	21.7	61.3	5.21
2	45.0	40.0	20.0	22.4	68.4	3.15
3	15.0	80.0	20.0	19.4	67.0	8.95
4	45.0	80.0	20.0	21.5	73.3	10.7
5	15.0	40.0	80.0	63.5	80.6	28.8
6	45.0	40.0	80.0	39.9	96.9	29.5
7	15.0	80.0	80.0	38.7	82.2	17.8
8	45.0	80.0	80.0	33.4	79.1	23.5
9	5.00	60.0	50.0	52.6	76.6	21.9
10	55.0	60.0	50.0	43.9	73.3	19.8
11	30.0	26.0	50.0	52.2	80.6	25.4
12	30.0	94.0	50.0	51.6	68.3	27.8
13	30.0	60.0	0.00	1.20	61.3	2.13
14	30.0	60.0	100	59.6	94.5	20.9
15	30.0	60.0	50.0	50.1	92.9	27.6
16	30.0	60.0	50.0	51.9	91.7	23.5
17	30.0	60.0	50.0	50.7	93.7	31.9

**Table 2:** Quadratic regression equations generated by CCD for the optimization of the UAE conditions of safflower seeds.

Response	Quadratic Regression Equation	* R^2^	** *p*
TAI (%)	Y_TAI_ = −31.60456 + 0.61586X_1_ + 0.50459X_2_ + 2.17259X_3_ + 8.20833 × 10^−3^X_1_X_2_ − 5.85417 × 10^−3^X_1_X_3_ − 8.80556 × 10^−3^X_2_X_3_ − 014449X_1_^2^ − 4.90116 × 10^−3^X_2_^2^ − 0.010971X_3_^2^	0.8823	0.0149
CAI (%)	Y_CAI_= −45.04668 + 2.20245X_1_ + 2.27937X_2_ + 1.20087X_3_ − 8.41667 ×10^−3^X_1_X_2_ − 5.55556 × 10^−3^X_1_X_3_ − 5.58333 × 10^−3^X_2_X_3_ − 0.026532X_1_^2^ − 0.015366X_2_^2^ − 5.59490 × 10^−3^X_3_^2^	0.9330	0.0024
RSA (%)	Y_RSA_ = −33.11801 + 0.52341X_1_ + 0.49596X_2_ + 1.27826X_3_ + 3.67083 × 10^−3^X_1_X_2_ + 1.86389 × 10^−3^X_2_X_3_ − 5.89375 × 10^−3^X_1_X_3_ − 0.013739 × 10^−3^X_1_^2^ − 2.64607 × 10^−3^X_2_^2^ − 7.28493 × 10^−3^X_3_^2^	0.9233	0.0038

* R^2^: coefficient of determination and ** *p*: probability value; *p*-value of < 0.05 indicates significance.

**Table 3:** ANOVA for the experimental results of the quadratic regression equations to the significance and adequacy of the models on the TAI, CAI, and RSA.

Variable	TAI (%)			CAI (%)			RSA (%)		
	Sum of Squares	F	*p*	Sum of Squares	F	*p*	Sum of Squares	F	*p*
Model	4157.32	5.84	0.0149	2066.32	10.83	0.0024	1397.69	9.37	0.0038
X_1_	121.48	1.53	0.2555	32.45	1.53	0.2559	0.48	0.029	0.8698
X_2_	92.33	1.17	0.3162	50.59	2.39	0.1663	0.21	0.012	0.9146
X_3_	2571.85	32.46	0.0007	1128.02	53.22	0.0002	771.91	46.56	0.0002
X_1_X_2_	48.51	0.61	0.4596	51.01	2.41	0.1648	9.70	0.59	0.4693
X_1_X_3_	125.61	1.59	0.2483	5.00 × 10^−3^	2.35 × 10^−4^	0.9882	5.63	0.34	0.5784
X_2_X_3_	98.70	1.25	0.3012	89.78	4.24	0.0786	100.04	6.03	0.0437
X_1_^2^	119.51	1.51	0.2589	403.24	19.02	0.0033	108.13	6.52	0.0379
X_2_^2^	43.49	0.55	0.4829	427.48	20.17	0.0028	12.68	0.76	0.4109
X_3_^2^	1052.51	13.28	0.0082	273.74	12.91	0.0088	464.09	27.99	0.0011

**Table 4:** Experimental values and coded levels of independent variables of CCD for optimization of the extraction condition of safflower seeds using UAE.

X_i_	Independent Variables	Coded Levels				
		−1.68	−1	0	+1	+1.68
X_1_	Extraction time (min)	5.00	15.0	30.0	45.0	55.0
X_2_	Extraction temperature (°C)	26.0	40.0	60.0	80.0	94.0
X_3_	Ethanol concentration (%)	0.00	20.0	50.0	80.0	100

## Data Availability

No new data were created or analyzed in this study. Data sharing does not apply to this article.

## Sample Availability

Samples of the compounds are not available from the authors.
